# Gait instability is a more specific predictor of corticospinal tract function than gait speed in clinically stable multiple sclerosis

**DOI:** 10.1038/s41598-025-10830-4

**Published:** 2025-07-23

**Authors:** Furkan Bilek, Arthur R. Chaves, Oleksandr Fesenko, Michelle Ploughman

**Affiliations:** 1 Recovery and Performance Laboratory, Division of Biomedical Sciences, Faculty of Medicine, Memorial University of Newfoundland, St. John’s, NL Canada; 2 Department of Gerontology, Fethiye Faculty of Health Sciences, Muğla Sıtkı Koçman University, Muğla, Turkey; 3 University of Ottawa’s The Royal Institute for Mental Health Research, Ottawa, ON Canada; 4 Interdisciplinary School of Health Sciences, Health Sciences Department, University of Ottawa, Ottawa, ON Canada

**Keywords:** Corticospinal tract, Spatiotemporal, Gait speed, Multiple sclerosis, Neurodegenerative disease, Gait stability, Transcranial magnetic stimulation, Neuroscience, Neurology

## Abstract

Multiple sclerosis (MS) research requires new, more sensitive, behavioral biomarkers that map to subtle central nervous system injury. Although gait speed, as measured using the Timed 25 Foot Walk Test, is used clinically to track MS progression, it is less useful in people with MS who do not have overt gait impairment. This study aimed to identify specific spatiotemporal gait parameters that predict corticospinal tract (CST) function in individuals with MS. We recruited consecutive patients attending a neurology clinic and evaluated CST excitatory and inhibitory function using single pulse transcranial magnetic stimulation of the primary motor cortex representation of the first dorsal interosseous muscle. We generated excitatory and inhibitory recruitment curves by calculating the area under the curve for motor-evoked potential amplitudes and cortical silent period durations, respectively, across stimulation intensities from 105 to 155% of active motor threshold in 10% increments. Spatiotemporal gait parameters were assessed using an electronic walkway. We built predictive models with gait parameters as the predictors and CST function as the outcome. We evaluated 78 individuals with MS (58 females). Longer distance of the center of pressure movement during single support was the strongest predictor of higher excitability (lower active motor threshold; accounting for 25.8% of variance, R² = 0.258), while less time in double support accounted for a smaller portion of variability in excitatory recruitment curve (13.3% variance explained, R² = 0.133). For inhibitory CST function, slower stride time (30.5% variance explained, R² = 0.305) and wider stride (6.3% variance explained, R² = 0.063) predicted greater inhibition. Notably, in all models, measures of gait stability, not gait speed, predicted CST function. Our results suggest that even among people with MS who have normal gait speed and can easily cross an urban intersection, subtle postural control impairments exist which may not be apparent to them or to their clinician.

**Supplementary Information:**

The online version contains supplementary material available at 10.1038/s41598-025-10830-4.

## Introduction

Human walking represents a remarkable neurobiological achievement - while appearing effortless in healthy individuals, it requires exquisitely timed sensorimotor integration to maintain dynamic balance during forward propulsion^1,2^. This continuous control of instability is why biomechanists describe walking as ‘controlled falling’, where minimal energy expenditure and automaticity mask the underlying complexity of neural control^1,3^. Optimal biomechanical alignment ensures efficiency, and spinal reflexes automatically control gait with input from visual, vestibular and sensory systems^4^. However, almost every person experiences gait dysfunction at one time or another^5^. The prevalence of gait impairment varies significantly by neurological condition and setting: from 60% in hospitalized neurological patients^6^, up to 93% in community-dwelling Parkinson’s disease with dementia patients^7^. This high prevalence is particularly notable in progressive multiple sclerosis, where gait dysfunction affects > 80% of patients^8^, underscoring its clinical significance. Because efficient gait requires smooth integration of the central and peripheral nervous systems with the musculoskeletal system, disrupted gait may originate from any of these making it difficult to ascertain whether changes in gait serve as potential biomarkers of degeneration of the central nervous system specifically^9^.

The corticospinal tract (CST) is the principal neural pathway regulating movement in humans^10,11^. In neurodegenerative diseases, changes in the CST are known to be associated with gait disturbances^12^. Specifically, in persons with MS, gait and balance disturbances are mainly associated with lesion load in the spinal cord^13,14^. For instance, shorter stance and longer time in double support were associated with diminished axonal fiber density and cross-sectional area of the CST in MS^15^. Hubbard et al. reported significant correlations between CST mean diffusivity—a diffusion metric sensitive to but not specific for myelin integrity, as it may also reflect axonal loss, gliosis, or inflammation—and functional gait measures (6-Minute Walk Test, Timed 25 Foot Walk Test, gait speed, and step length)^16^. Using 7 T magnetic resonance imaging, Lizama and group reported that greater CST axonal loss, but not changes in cerebellar-thalamic tracts, was associated with gait instability measured on a treadmill^17^. However, there exists a clinico-radiologic paradox in which structural imaging findings often correlate poorly with clinical outcomes^18–20^. Accumulating evidence suggests that inflammatory central nervous system lesions on brain imaging are transient and symptoms are more associated with disruption in functional brain networks that are not always well localized to individual brain structures^21–24^. In contrast to the localization of structural MS-related foci, Transcranial Magnetic Stimulation (TMS) measures CST function in real-time^25–27^. Among people with MS, TMS studies report increases in CST motor-evoked response latency and lowered magnitude^24^, increased resting motor threshold (lower excitability) and prolonged silent period (greater inhibition)^28^. Motor evoked potentials (MEPs) elicited using TMS over the primary motor cortex represent a valuable method for assessing CST function, perhaps before CST damage become visible with structural imaging^29^. Prodromal MS, progression in the absence of relapse activity - PIRA, Radiologically-Isolated Syndrome and Clinically Isolated Syndrome are disease states in which ‘covert’ or subclinical neurodegeneration takes place^30,31^. These newly recognized disease states and the development of high-efficacy medications to dampen relapses require new approaches to detect subclinical neurodegeneration and subsequent, often covert, gait impairment.

We undertook this study to test the degree to which spatiotemporal gait parameters would predict CST function as measured using TMS in a sample of persons with MS. Recognizing that the most commonly utilized gait measure in MS records the time to walk 25 ft as quickly as possible using a stopwatch^32^, we hypothesized that spatiotemporal parameters extracted from a pressure-sensitive electronic walkway would yield more sensitive prediction of CST function.

## Materials and methods

### Participants

We conducted the study according to the Declaration of Helsinki guidelines and the approval of the Health Research Ethics Board (HREB# 2015.103). All participants provided written informed consent for the study. After obtaining informed written consent, we recruited consecutive patients attending a specialized MS clinic. Inclusion criteria were ≥ 18 years of age, a definite diagnosis of MS according to the McDonald criteria^33^the ability to walk at least 100 m without a gait aid, and inactive and relapse-free disease for ≥ 3 months. We excluded participants with severe balance or gait disturbances, participants with contraindications for TMS^34^and participants unable to complete the entire assessment.

### Study design and procedure

This cross-sectional study took place on a single session. Participants first underwent TMS testing and completed gait analysis. We collected age, sex, MS type, disease duration, disability status (Expanded Disability Status Scale [EDSS]), comorbidities, and medications from health records. Since we intended to use regression modeling to determine the spatiotemporal variables with greatest predictive value in terms of CST function, we considered the “rule of tens,” a statistical principle that recommends the number of variables that can be predicted from a data set; a widely utilized approach in traditional clinical predictive modeling strategies^35,36^. Given our intention to include four to six gait variables in the regression model, the requisite sample size should be at least 60, in accordance with this rule^37^.

### Transcranial magnetic stimulation

We assessed CST function by stimulating the motor cortex area corresponding to the first dorsal interosseous muscle of the weaker hand using TMS^29^. We chose this muscle based on its large representation in the motor cortex and our previous work confirming our ability to obtain a MEP even among people with very low CST excitability as well as the relationship between TMS outputs derived from the hand and gait function^29,38–40^. We first identified the weaker hand using a calibrated dynamometer (Lafayette Instrument Corp., Lafayette, IN, USA)^29,40^. A BiStim 200^2^ magnetic stimulator, connected to a 70 mm figure-of-eight coil (Magstim Co. Whitland, UK), delivered monophasic pulses. We positioned the coil tangential to the scalp and directed the handle at a 45° angle posterolateral to the midsagittal line, thus generating a posterior-anterior flow^26^. We positioned electromyographic recording electrodes (Coviden, Mansfield, MA, USA) over the first dorsal interosseous. We determined the hotspot; the site that elicited the largest MEPs after a series of suprathreshold TMS pulses, marked using neuronavigation (Brainsight™, Rogue Research, Montréal, QC, Canada)^29^. We used the same system to collect electromyographic muscle activity and record MEPs with the internal electromyographic system.

Our previously published detailed protocol outlines the systematic approach to obtaining MEPs, determining the hotspot and motor thresholds as well as how we calculated the recruitment curves^29^. In brief, we defined the active motor threshold (AMT) as the minimum stimulation intensity expressed as a percentage of the maximum stimulus output required to produce MEPs with a peak-to-peak amplitude of at least 200 µV on at least five out of ten trials during 10% of maximal voluntary pinch^26^. AMT assesses the glutamate-mediated excitability of low-threshold neurons and serves as a reflection of the intrinsic excitability of the motor cortical representation^26,41^. In MS, a reduction in AMT is indicative of demyelination and axonal damage^42^. Subsequently, we collected data from excitatory (eREC) and inhibitory (iREC) MEP recruitment curves. During 10% maximal tonic contraction, we elicited MEPs from the hemisphere contralateral to the weaker hand. We performed six stimulation trials (36 in total), each at 105–155% of AMT, in 10% increments, in a randomized order, with trial intervals of 4–10 s with a short rest between blocks. We generated eREC and iREC MEP curves based on MEP amplitude (µV) and cortical silent period duration (ms) versus stimulus intensity (%AMT). MEP recruitment curves characterize the input-output properties of corticospinal pyramidal neurons and inhibitory interneurons^26,42^.

### Spatiotemporal gait parameters

The Zeno Walkway platform (ProtoKinetics Havertown, PA) records plantar pressures and gait parameters during ambulation. The Zeno Walkway platform measures 4.2 m in length × 0.9 m in width, comprising 36,864 pressure sensors sampled at 120 Hz. We used the Protokinetics Movement Analysis Software - PKMAS (ProtoKinetics, Havertown, PA) to extract spatial, temporal and stability parameters (Table S1)^43–46^. Each participant walked as quickly but safely as possible without running (fast walking). They completed two trials on the instrumented walkway; commencing 1 m before the end of the walkway and returning 1 m after the end of the walkway.

### Stability-related gait parameters

To evaluate covert balance impairments and dynamic gait stability, we extracted several stability-related parameters from the instrumented walkway system^43–46^. These included step time coefficient of variation and stride length coefficient of variation, which are widely accepted measures of gait variability and have been associated with postural control deficits and increased fall risk in both older adults and people with neurological disorders, including multiple sclerosis^9,46,47^. Temporal and spatial gait variability reflect irregular motor control and are linked to diminished central nervous system adaptability^48,49^. We also examined center of pressure distance during stance, single support and double support phases. These parameters capture the anteroposterior displacement of the body’s center of mass relative to the base of support, serving as proxies for balance confidence and efficiency. Reduced excursion is associated with cautious gait patterns and impaired postural response^44,47,49^. Furthermore, we calculated center of pressure path efficiency during single support and double support phases, representing the linearity of center of pressure trajectories. Lower efficiency indicates increased corrective movements and impaired dynamic balance control^9,50^. These center of pressure-based and variability metrics, although distinct from nonlinear dynamic stability indices such as Lyapunov exponents or harmonic ratios, are highly feasible in clinical settings and provide valid insights into subtle motor control dysfunctions^50,51^. Although variability metrics such as stride length coefficient of variation or step time coefficient of variation are not direct indices of dynamic stability (e.g., as compared to Lyapunov exponents), they serve as indicators of central nervous system dysregulation impacting gait consistency—an important component of functional stability^52–54^.

The selection of gait stability parameters—including center of pressure distance and stride time/length variability—was specifically tailored to align with the neurophysiological mechanisms underlying MS^17^. The increased center of pressure distance during single support phase reflects efficient central postural control mediated by CST integrity^9,47^while prolonged double support time reveals compensatory balance strategies associated with CST neurophysiological indicators of altered CST function^15^. Similarly, stride time, step time etc. metrics correlate with CST damage^17^. Unlike harmonic ratio or Lyapunov exponents, which assess rhythmicity or system stability, our chosen parameters capture phase-specific gait dynamics that directly reflect CST-mediated control. This approach provides greater sensitivity to neurodegenerative processes in MS and offers clinically meaningful insights into gait instability.

### Statistical analysis

We considered three groups of gait variables (spatial, temporal and stability [Table S1]) and variables proceeded through the regression steps in these groups. We used the statistical program SPSS 28.0 for all analyses. We defined the significance alpha level at < 0.05. The normality of the dependent variable was verified using the Shapiro–Wilk test. All data were normally distributed. First, we used Pearson correlation analysis to examine the relationships between TMS and gait data. Correlations were considered negligible between 0 and 0.20, weak if 0.21–0.40, moderate if 0.41–0.60, strong if > 0.61^55^. Although we classified correlation strength (e.g., weak, moderate, strong) to aid interpretation of effect size, only statistically significant correlations (*p* < 0.05), regardless of their r-value magnitude, were entered into regression modeling. Only those with significant relationships proceed to the next regression-modelling phase. To identify which of the gait parameters best predicted TMS outcomes, we conducted a three-step regression analysis. In the initial step, we used simple linear regression to assess the predictive value of the independent variables that were identified as significantly correlated with the dependent variables (iREC, eREC, AMT). In step 2, these variables which predicted variability in TMS data were entered into hierarchical regression within their respective groups (spatial, temporal, stability) ordered from highest to lowest R² values derived from step 1. Before commencing step 3, as a consequence of the analysis conducted in step 2, only those predictors that significantly contributed to outcome variability were entered into a final hierarchical regression, similar to step 2, irrespective of gait variable group (Table S1). In order to determine the validity of predictive models in participant subgroups with and without structural CST lesions, most recent magnetic resonance imaging data and reports were obtained from health records. Reports and images were inspected for lesions in the corticospinal tract, from the cortical surface to the spinal cord, and coded as “Yes” or “No”. Predictive gait variables that were identified in the full sample were then tested in the subgroups (CST structural lesions “Yes” or “No”).

Some of the contents of this manuscript was presented in poster format at the Neuroscience 2024 conference in Chicago, USA, from October 5-9, 2024.

## Results

### Participant characteristics

We recruited 105 participants but due to incomplete data, 27 were excluded, leaving 58 females and 20 males. The most common reason for incomplete data was that the thresholds required to obtain a MEP were beyond the capacity of the TMS system. The mean age of the participants was 47.91 ± 10.15 years (range: 21–70). The median EDSS score was 2.0 (interquartile range: 1.5) and average fast walking speed was 178 cm/s; both values suggest participants had minimal MS-related disability^33^ and would be able to successfully cross a typical urban intersection^56^. Sixty-nine participants had relapsing-remitting MS, eight had secondary progressive MS and one had primary progressive MS (Table 1). 

**Table 1:** Demographic and clinical characteristics of the participants.

Sex (*n*)	58 Female, 20 Male
Age (years)	47.91 ± 10.15
BMI (kg/m^2^)	28.60 ± 6.67
MS Type	69 RRMS, 8 SPMS, 1 PPMS
Disease Duration (years)	14.29 ± 8.19
Velocity (cm/sec)	178.05 ± 53.39
MS Severity (EDSS 0–10)	Median: 2; Interquartile range: 1.5 Minimum/Maximum: 0/6.5
Dominant Side (right/left)	68 Right, 9 Left
Weaker Side (dominant/nondominant)	23 Dominant, 55 Nondominant

BMI: body mass index; kg: kilogram; m: meter; MS: Multiple Sclerosis; EDSS: Expanded Disability Status Scale; RRMS: relapsing-remitting MS; SPMS: secondary progressive MS; PPMS: primary progressive MS.

### Gait stability, not speed, predicted better corticospinal tract function

Slower gait speed, shorter step lengths and other indicators of worse gait were related to altered function of the CST (higher motor threshold [less excitability] and greater inhibition) (*p* < 0.05, Table S2). While initial bivariate analyses revealed significant correlations between gait speed and CST function measures (AMT: *r* = −0.493, *p* < 0.001; eREC: *r* = 0.341, *p* = 0.002; iREC: *r* = −0.479, *p* < 0.001) (Table S2), these relationships were attenuated in regression models accounting for other spatiotemporal gait parameters. This suggests in simple linear regression model, gait speed shares substantial variance in predicting CST function (AMT: R²=0.243, *p* < 0.001; eREC: R²=0.117, *p* = 0.003; iREC: R²=0.229, *p* < 0.001) (Tables 2, 3 and 4). The second and third hierarchical regression steps prioritized variables explaining unique variance, revealing that stability parameters were more specific predictors of CST neurophysiology than gait speed alone (Tables 2, 3 and 4).

**Table 2:** Predictors of transcranial magnetic stimulation-derived active motor threshold of the corticospinal tract.

		Results of Simple Linear			Results of Within Group Stepwise		Results of Without Group Stepwise	
DV is AMT	Independent variable	R	R^2^	p	ΔR^2^​​	p	ΔR^2^​​	p
Spatial	Stride Length (cm)	0.410	0.168	0.000214**	0.168	0.000214**		
	Step Length (cm)	0.409	0.168	0.000220**				
	Absolute Step Length (cm)	0.389	0.151	0.000471**				
	Stride Width (cm)	0.355	0.126	0.001520**	0.058	0.021185*		
Temporal	Total Double Support (%)	0.499	0.249	0.000005**	0.249	0.000005**		
	Stride Time (sec)	0.486	0.236	0.000007**				
	Velocity (cm/sec)	0.493	0.243	0.000005**				
	Single Support (%)	0.490	0.240	0.000008**				
	Cadence (steps/min)	0.440	0.194	0.000062**				
	Swing Time (sec)	0.387	0.150	0.000500**				
Stability	SS COP Distance (cm)	0.516	0.266	0.000002**	0.266	0.000002**	0.258	0.000003**
	DS COP Distance (cm)	0.256	0.065	0.024774*				
	Stride Length CV (cm)	0.236	0.056	0.038487*				

Step 1 includes only those predictors that were significantly correlated with the outcome. Variable significance in step 1 is proceeded to stepwise regression. DV: dependent variable; AMT: active motor threshold; cm: centimeter; sec: seconds; CV: coefficient of variation; SS: single support; DS: double support; COP: center of pressure; p: confidence interval *p < 0.05; **p < 0.01.

**Table 3:** Predictors of transcranial magnetic stimulation-derived excitatory recruitment curve of the corticospinal tract.

		Results of Simple Linear			Results of Within Group Stepwise		Results of Without Group Stepwise	
DV is eREC	Independent variable	R	R^2^	p	Δ R^2^​​	P	Δ R^2^​​	p
Spatial	Stride Length (cm)	0.309	0.095	0.007413**	0.095	0.007413**		
	Step Length (cm)	0.304	0.093	0.008367**				
	Absolute Step Length (cm)	0.278	0.077	0.016423*				
Temporal	Total Double Support (%)	0.364	0.133	0.001648**	0.133	0.001648**	0.133	0.001648**
	Single Support (%)	0.358	0.128	0.002041**				
	Velocity (cm/sec)	0.342	0.117	0.002906**				
	Stride Time (sec)	0.324	0.105	0.004819**				
	Cadence (steps/min)	0.298	0.089	0.009848**				
	Swing Time (sec)	0.230	0.053	0.049084*				
Stability	DS COP Path Efficiency (%)	0.331	0.110	0.003960**	0.110	0.003960**		
	SS COP Distance (cm)	0.320	0.102	0.005516**				
	DS COP Distance (cm)	0.241	0.058	0.038434*				

Step 1 includes only those predictors that were significantly correlated with the outcome. Variable significance in step 1 is proceeded to stepwise regression. DV: dependent variable; eREC: excitatory recruitment curve; cm: centimeter; sec: seconds; CV: coefficient of variation; SS: single support; DS: double support; COP: center of pressure; p: confidence interval *p < 0.05; **p < 0.01.

In the second step of our predictive models, we identified which gait variables were the most predictive within their respective groups when considered in the context of the TMS data. In terms of the TMS variable, AMT (a measure of excitability), higher excitability (lower AMT) was predicted by two spatial variables, longer stride (16.8%) and narrower stride (5.8%), one temporal variable, shorter time in double support (24.9%) and one stability variable, longer center of pressure excursion during single support (26.6%) (Table 2). Regarding the TMS variable eREC (a measure of excitability), higher excitability (greater eREC) was predicted by one spatial variable, longer stride length (9.5%), one temporal variable, shorter time in double support (13.3%) and one stability variable, increased path efficiency during double support (11.0%) (Table 3). In consideration of the TMS variable iREC (a measure of inhibition), higher inhibition (higher iREC) was predicted by two spatial variables, wider stride (21.3%) and shorter step length (7.0%), two temporal variables, longer time in double support (5.7%) and increased stride time (30.9%) and two stability variables, shorter center of pressure excursion during single support (24.9%) and poorer double support path efficiency (5.5%) (Table 4).

**Table 4:** Predictors of transcranial magnetic stimulation-derived inhibitory recruitment curve of the corticospinal tract

		Results of Simple Linear			Results of Within Group Stepwise		Results of Without Group Stepwise	
DV is iREC	Independent variable	R	R^2^	p	Δ R^2^	p	Δ R^2^​​	p
Spatial	Stride Width (cm)	0.461	0.213	0.000058**	0.213	0.000058**	0.063	0.012994*
	Stride Length (cm)	0.374	0.140	0.001425**				
	Step Length (cm)	0.373	0.139	0.001477**	0.070	0.012950*		
	Absolute Step Length (cm)	0.336	0.113	0.004500**				
Temporal	Total Double Support (%)	0.552	0.305	0.000001**	0.057	0.032714*		
	Stride Time (sec)	0.542	0.293	0.000001**	0.309	0.000008**	0.305	0.000001**
	Single Support (%)	0.541	0.293	0.000002**				
	Cadence (steps/min)	0.497	0.247	0.000012**				
	Velocity (cm/sec)	0.479	0.229	0.000028**				
	Swing Time (sec)	0.422	0.178	0.000275**				
Stability	SS COP Distance (cm)	0.499	0.249	0.000011**	0.249	0.000011**		
	DS COP Path Efficiency (%)	0.484	0.235	0.000022**	0.055	0.025117*		
	Stride Length CV (%)	0.247	0.061	0.039582*				

Step 1 includes only those predictors that were significantly correlated with the outcome. Variable significant in step 1 proceeds to stepwise regression. DV: dependent variable; iREC: inhibitory recruitment curve; cm: centimeter; sec: seconds; CV: coefficient of variation; SS: single support; DS: double support; COP: center of pressure; p: confidence interval *p < 0.05; **p < 0.01.

**Fig. 1:**
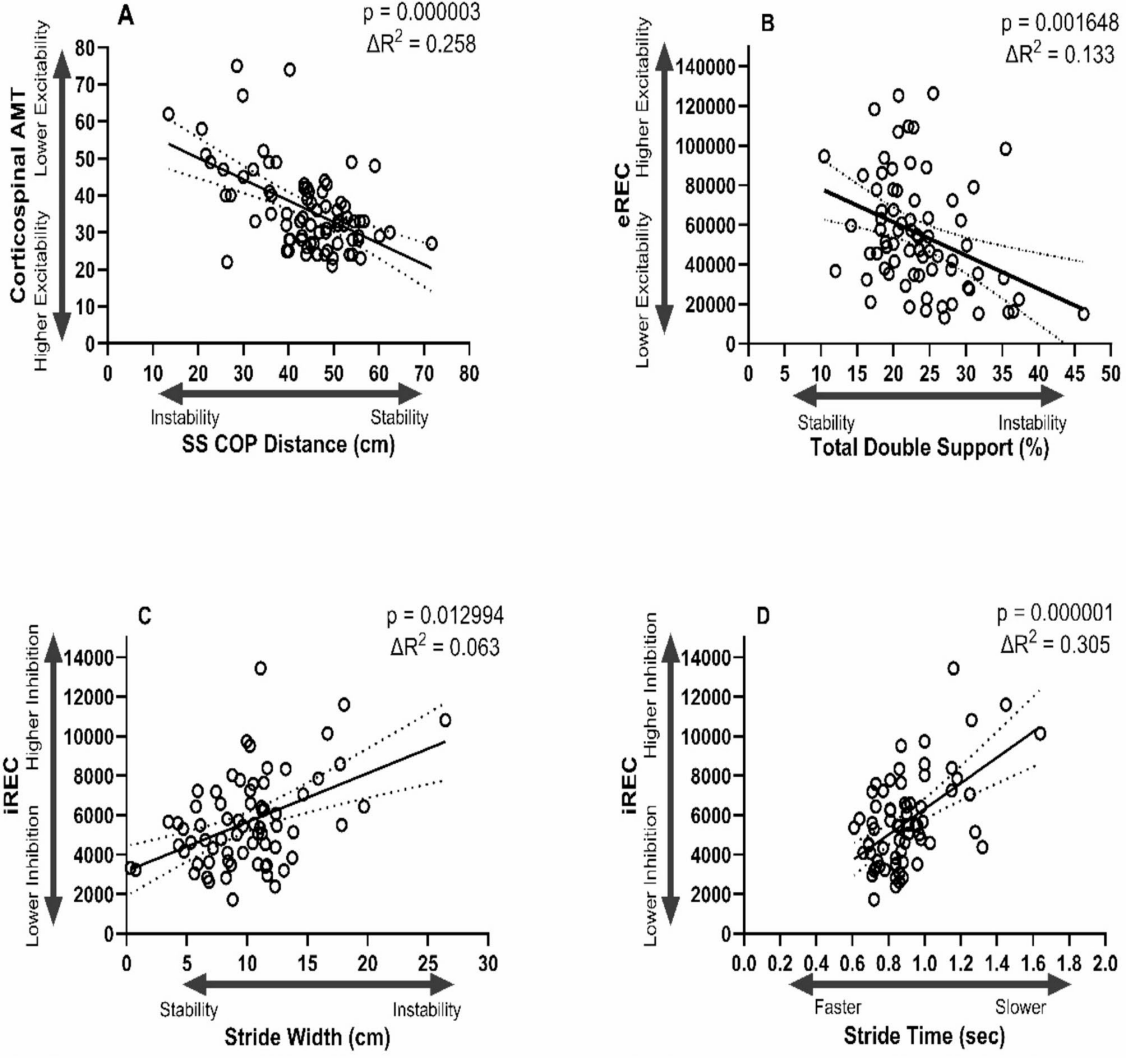
Regression plots of gait parameters and corticospinal tract (CST) function. **A** CST excitability and single support center of pressure distance. **B** CST excitability and total double support. **C** CST inhibition and stride width. **D** CST inhibition and stride time. AMT: active motor threshold; eREC: excitatory motor evoked potential recruitment curve; iREC: inhibitory motor evoked potential recruitment curve; SS: single support; COP: center of pressure. Note: The dotted lines represent the 95% confidence intervals of the regression line.

In the final predictive model, we included those gait variables (without grouping) that were most predictive in the previous steps. In this final step, the sole parameter that predicted lower AMT (higher excitability) was longer single support center of pressure distance (25.8%), which is an indicator of efficient balance and stability (Table 2; Fig. 1A). The sole parameter that predicted higher eREC (higher excitability) was less time in total double support (13.3%) which is also an indicator of better balance and stability (Table 3; Fig. 1B). The gait parameters that predicted higher CST inhibition (indicating higher iREC) were wider stride (6.3%) (Table 4; Fig. 1C) and increased stride time (30.5%) (Table 4; Fig. 1D). The final predictors of CST function in the total sample remained statistically significant in the subgroups with and without CST structural lesions (identified using structural imaging), with the exception of percentage time in double support/eREC in the No-lesion group (Table 5).

**Table 5:** Validation analysis in subgroups with and without CST structural lesions.

Predictive model	Total Sample	CST Lesion Subgroup	CST No-Lesion Subgroup
	*R* ^2^	*R* ^2^	*R* ^2^
SS COP Distance (cm) - AMT	0.258*	0.254*	0.444*
Total Double Support (%) - eREC	0.133*	0.210*	0.037
Stride Width (cm) - iREC	0.063*	0.431*	0.137*
Stride Time (sec) - iREC	0.305*	0.230*	0.429*

AMT: active motor threshold; eREC: excitatory motor evoked potential recruitment curve; iREC: inhibitory motor evoked potential recruitment curve; cm: centimeter; sec: seconds; SS: single support; COP: center of pressure; CST: corticospinal tract; R^2^: coefficient of determination; p: confidence interval *p < 0.05.

## Discussion

We undertook this study in order to determine, among a clinic sample of persons with MS, which gait spatiotemporal parameters predicted (covert) functional changes in CST, a critical neural pathway controlling voluntary movement. We considered both the excitatory (activation threshold of surface neurons in the motor cortex) and inhibitory (GABA-mediated intracortical inhibition) functions of the CST^29,57^. We report three main findings. First, although gait speed (as part of the Timed 25 Foot Walk Test), is the most common gait outcome in MS, gait speed was not predictive of excitatory or inhibitory CST function. Secondly, when considering predictors of excitatory CST function, longer distance of the center of pressure during single support was the strongest predictor of higher excitability (lower AMT); accounting for 25.8% of the variability. Less percentage of time in double support accounted for a significant but small (13.3%) degree of variability in eREC (higher excitability). Finally, in terms of inhibitory function of the CST, slower stride time (30.5%) and to a lesser degree, wider stride (6.3%) accounted for the variability in iREC (greater inhibition).

Gait speed is a commonly utilized clinical measure for assessing and predicting the level of disability in neurodegenerative diseases^46,58,59^. However, our findings indicate that speed showed strong bivariate correlations with CST function, its predictive value was largely subsumed by more specific stability measures in multivariate models. Indeed, a number of factors, including balance, fatigue, lower limb muscle weakness, and cardiorespiratory fitness, can affect gait speed^59–63^ and are likely prerequisites for fast walking^64^. The contributions of the skeletal, muscular and cardiovascular systems to gait make it difficult for clinicians to decipher whether slowing of gait speed is due to deterioration of the central nervous system specifically. Changes in gait speed are also not particularly sensitive; requiring a change of about 20% to be considered meaningful in people with stable disease^65^. Our results support the use of more sophisticated digital tools to monitor gait and balance changes such as those employed by NIH Toolbox and other electronic methodologies^66^. Clinically, that while gait speed remains a valuable global indicator, we recommend assessing gait stability (total double support, double support center of pressure distance, stride width), which related to the function of the CST (as measured using TMS), than gait speed, particularly during prodromal MS or PIRA. In this context, although variability-based gait metrics are not direct measures of dynamic stability in the biomechanical sense, they offer insight into the consistency and automaticity of gait control^52,67–69^. These fluctuations reflect reduced efficiency in central motor planning and increased reliance on conscious postural strategies—particularly relevant in early or covert CST dysfunction^68,70–72^. Thus, although their initial use has often been tied to fall risk, recent evidence highlights their utility as markers of central dysregulation that undermines functional gait stability, even in individuals with normal walking speed^70,73,74^.

CST excitability is governed by CST size, the immediate availability of glutamatergic neuronal pools and influences of descending reticulospinal pathways^75^. In our MS cohort, gait instability measures (shorter single support center of pressure distance and longer time in double support) were associated with neurophysiological indicators of altered CST function^76^. These two gait stability parameters, single support center of pressure distance and double support, are calculated from the center of pressure; the point of application for all forces exerted on the surface during foot-ground contact, with its movement being dependent on the dynamics of the body’s center of mass^47^. In the healthy central nervous system with intact postural control, the center of pressure excursion under the foot is long because the person walking is able to automatically adjust for displacement of their center of gravity as they quickly and efficiently move forward. This confidence affords them the ability to spend more time with only one foot on contact with the ground (single support) as opposed to two (double support). In aging and neurodegenerative diseases, covert postural control impairments result in the person restricting excursion of the body and subsequently the center of mass; resulting in a protective slowing of gait with reduced center of pressure excursion^77^. Our results suggest that even among people with MS who have normal gait speed and can easily cross an urban intersection, subtle postural control impairments exist which may not be apparent to them or to their clinician. We argue, along with others, that disrupted postural control likely precedes slowing of gait^78–80^; the challenge is that such subtle impairment is difficult to measure using conventional tools such as stopwatches or ordinal grading systems like those used in the Berg Balance Scale or Dynamic Gait Index. We propose that persons with subclinical/emerging postural control impairments should be offered preventative rehabilitation and agility training to forestall gait impairment employing digital/electronic outcome measures that index CST function^39^gait, postural control and agility^77,81^.

Control of human movement requires not only excitation of motor circuits but appropriate control processes that modify, suppress or even cancel motor evoked potentials^82,83^. The CST is not simply a motor execution pathway; it also modulates (via inhibition) proprioceptive input ascending in the spinal cord^11,84^ and receives intracortical inhibitory input from adjacent cortex^11^. Our previous work suggests that people with MS exhibit inappropriate suppression (disinhibition) of inhibition, which was associated with weaker hand strength^85^. However, we also showed that in terms measurement of the cortical silent period that follows a MEP, an indicator of CST inhibition, excessive quiescence (slow return of typical background electromyographic activity) was linked to slower walking speed^29^lower fitness and greater fatigue among persons with MS^86^. The results of the current work confirm that slower stride time (30.5%) and to a lesser degree, wider stride (6.3%) predicted greater CST inhibition (iREC). Other evidence indicates that higher inhibition was associated with a reduction in the activity of the leg muscles during the gait cycle^87^even among healthy young adults^88^. Suppressed leg muscle activity would likely increase stride time (defined as the interval between two consecutive heels strikes by the same leg); providing greater stability but also slowing gait speed^89–91^. Participants with greater inhibition of the CST also exhibited wider stride. Widening the stride would compensate for feelings of instability that would likely be transmitted via proprioceptive spinal inputs to the CST^92^. Our results confirm that functional network changes, likely from spinal cord afferent and within the cortex, likely precede structural damage visible on diagnostic imaging. These covert excitatory and inhibitory alterations affect dynamic gait and balance and are a potential target for reparative therapies^39,93^.

Although we report a comprehensive assessment of preclinical gait impairments and its relationship to CST function, the study has some limitations. First, even though we validated our models in subgroups with and without structural CST lesions, other central nervous system regions such as the cerebellum could affect the CST function and dynamic gait. Secondly, anthropometric characteristics may exert an influence on gait speed. For example, individuals with longer legs may be more likely to walk at a faster pace^94^. The high representation of female participants increases the likelihood that the data reflect the female’s gait parameters. It would be beneficial for future research to consider sex and anthropometric differences^95^. Third, the median EDSS score was 2 with a narrow interquartile range and the majority of participants had a diagnosis of relapsing-remitting MS and normal gait speed, which limits the generalizability of our findings to those who have mild or no walking disability. Fourthly, another limitation of the study is its cross-sectional design. Long-term follow-up would be necessary to observe parallel changes in gait analysis and TMS measurements. Fifthly, the hierarchical regression approach, while appropriate for our hypothesis testing, may obscure relationships between highly correlated variables. Future studies could employ alternative approaches (e.g., factor analysis or machine learning) to better disentangle shared versus unique variance among gait parameters. Finally, our TMS measures assessed CST projections to hand muscles rather than leg muscles directly involved in gait. While we and others have demonstrated relationships between upper limb TMS measures and lower limb function in MS, future studies should incorporate lower limb motor cortex stimulation to confirm these findings.

## Conclusion

Our findings reveal that gait stability parameters significantly relate to corticospinal tract neurophysiology in clinically stable MS patients with preserved walking speed (mean 178 cm/s). In individuals with MS with no overt gait problems, measures of gait stability were stronger predictors of CST function than slowing of gait speed. A longer center of pressure distance during single support (ability to comfortably/confidently advance the body’s center of mass forward while on one foot) was associated with higher CST excitability; while a greater percentage of time spent in double support (maintaining both feet on the ground to obtain greater stability) was associated with lower CST excitability; longer stride duration and wider stride were associated with greater CST inhibition. The findings suggest that the use of more sophisticated digital tools, rather than manual timing of gait with stopwatches, may be a more effective method for detecting these covert dynamic gait changes that likely precede slowing of gait. Digital tools that assess gait stability, rather than gait speed, will help in the development of rehabilitation interventions to challenge agility and postural control in order to promote neuroplasticity and potentially forestall declines in gait speed.

## Electronic supplementary material

Below is the link to the electronic supplementary material.

Supplementary Material 1

## Data Availability

The datasets used and/or analyzed during the current study are available from the corresponding author on reasonable request.
