# Supplementary material for: Gait instability is a more specific predictor of corticospinal tract function than gait speed in clinically stable multiple sclerosis
Source: Sci Rep. 2025 Jul 23;15:26822. doi: 10.1038/s41598-025-10830-4 (PMC12287266; doi:10.1038/s41598-025-10830-4)
Supplement: Supplementary file 1 — Supplementary Material 1 [file 41598_2025_10830_MOESM1_ESM.docx]

**Supplementary files**

Supplementary Table S1

| **Table S1.** Spatiotemporal gait parameters. | | | |
| --- | --- | --- | --- |
|  | Variable | Description | Clinical Significance: In neurodegenerative diseases |
| Spatial | Step Length | Step Length is the distance between corresponding successive heel points of opposite feet, measured parallel to the direction of progression for the ipsilateral stride (cm). | Decreased |
|  | Absolute Step Length | The diagonal distance between successive points on the heel of opposite feet, measured for the stride of which it is the second part (cm). | Decreased |
|  | Stride Length | The distance from the heel of one foot to the following heel of the same foot (cm). | Decreased |
|  | Stride Width | Stride Width is the perpendicular distance between the line connecting the two ipsilateral foot heel contacts (stride) with the contralateral heel contact between those events (cm). | Increased |
| Temporal | Stride Time | Stride time is the period of time from first contact of one foot, to the following first contact of the same foot (sec). | Decreased |
|  | Swing Time | The period of time the foot is not in contact with the ground (secs). | Decreased |
|  | Single Support | The period of time when only the current foot is in contact with the ground. Single Support Time expressed as a percentage of the Gait Cycle Time (%). | Increased |
|  | Total Double Support | The sum of all periods when both feet are in contact with the ground. Total Double Support Time presented as a percentage of the Gait Cycle Time (%) | Increased |
|  | Velocity/Gait Speed | The Velocity is measured by dividing the sum of all Stride Length measurements by the sum of all Stride Time measurements of both feet (cm/sec). | Decreased |
|  | Cadence | The number of footfalls minus one divided by the ambulation time, converted to minutes (steps/min). | Increased |
| Stability | Step Time CV | The period of time taken for one step measured from first contact of one foot to the first contact of following other foot (sec). The CV divides the Standard Deviation by the Mean and presents the value as a percent of 100 (%). | Decreased |
|  | Stride Length CV | The distance from the heel of one foot to the following heel of the same foot (cm). The CV divides the Standard Deviation by the Mean and presents the value as a percent of 100 (%). | Decreased |
|  | Stance COP Distance | The direct distance between first and last contact of the COP Left or Right waveform during stance phase (cm). | Increased |
|  | SS COP Distance | The direct distance between the COP Left or Right waveform during single support phase (cm). | Increased |
|  | DS COP Distance | The direct distance between for the COP Both waveform during double support phase (cm). | Increased |
|  | SS COP Path Efficiency | Single Support COP Path Efficiency measures the COP Left or Right start to end distance during single support as a percent of the COP Path length of the same footfall during single support phase (%). | Decreased |
|  | DS COP Path Efficiency | Double Support COP Path Efficiency measures the COP Left or Right start to end distance during double support as a percent of the COP Path length of the same footfall during the double support phase (%). | Decreased |
| cm: centimeter; sec: seconds; CV: coefficient of variation; SS: single support; DS: double support; COP: center of pressure. | | | |

Supplementary Table S2

| **Table S2.** Correlations between transcranial magnetic stimulation-derived variables and spatiotemporal and stability parameters | | | | | | | |
| --- | --- | --- | --- | --- | --- | --- | --- |
|  |  | AMT | | eREC | | iREC | |
|  |  | r | p | r | p | r | p |
| Spatial | Step Length (cm) | -0.409 | 0.000219** | 0.304 | 0.008367** | -0.373 | 0.001477** |
|  | Absolute Step Length (cm) | -0.389 | 0.000471** | 0.278 | 0.016423* | -0.336 | 0.004500** |
|  | Stride Length (cm) | -0.410 | 0.000214** | 0.309 | 0.007413** | -0.374 | 0.001425** |
|  | Stride Width (cm) | 0.355 | 0.001520** | -0.049 | 0.680189 | 0.461 | 0.000058** |
| Temporal | Stride Time (sec) | 0.486 | 0.000007** | -0.324 | 0.004819** | 0.542 | 0.000001** |
|  | Swing Time (sec) | 0.387 | 0.000500* | -0.230 | 0.049084* | 0.422 | 0.000275** |
|  | Single Support (%) | -0.490 | 0.000008** | 0.358 | 0.002041** | -0.541 | 0.000002** |
|  | Total Double Support (%) | 0.499 | 0.000005** | -0.364 | 0.001648** | 0.552 | 0.000001** |
|  | Velocity (cm/sec) | -0.493 | 0.000005** | 0.341 | 0.002906** | -0.479 | 0.000028** |
|  | Cadence (steps/min) | -0.440 | 0.000062** | 0.298 | 0.009848* | -0.497 | 0.000012** |
| Stability | Step Time CV (%) | 0.019 | 0.872402 | -0.252 | 0.031307 | 0.156 | 0.201584 |
|  | Stride Length CV (%) | 0.236 | 0.038427* | -0.031 | 0.794558 | 0.247 | 0.039582* |
|  | Stance COP Distance (cm) | -0.218 | 0.056972 | 0.151 | 0.199131 | -0.094 | 0.437062 |
|  | SS COP Distance (cm) | -0.516 | 0.000002** | 0.320 | 0.005516** | -0.499 | 0.000011** |
|  | DS COP Distance (cm) | -0.256 | 0.024774* | 0.241 | 0.038434* | -0.129 | 0.288386 |
|  | SS COP Path Efficiency (%) | 0.078 | 0.498607 | 0.157 | 0.182068 | 0.016 | 0.898385 |
|  | DS COP Path Efficiency (%) | 0.116 | 0.315579 | 0.331 | 0.003960** | -0.484 | 0.000022** |
| AMT: active motor threshold; eREC: excitatory motor evoked potential recruitment curve; iREC: inhibitory motor evoked potential recruitment curve; cm: centimeter; sec: seconds; CV: coefficient of variation; SS: single support; DS: double support; COP: center of pressure; r: pearson’s correlation coefficient; p: confidence interval *p < 0.05; **p < 0.01. | | | | | | | |
